# Perceptions and Interests of Dental Hygienists in Addressing Food Insecurity and HPV in Clinical Settings

**DOI:** 10.1111/jphd.70030

**Published:** 2026-01-25

**Authors:** Tuba Khan, Abby Fleming, Jillian M. Joyce, Kathleen J. Porter, Rachel A. Liebe

**Affiliations:** ^1^ Department of Nutritional Sciences, College of Education and Human Sciences Oklahoma State University Stillwater Oklahoma USA; ^2^ Department of Public Health Sciences, School of Medicine University of Virginia Christiansburg Virginia USA

**Keywords:** dental hygienists, food insecurity, health professional education, human papillomavirus, social/behavioral change

## Abstract

**Purpose:**

Few studies have explored dental hygienists' views on health behaviors like food insecurity or recommending preventive care such as the HPV vaccine. This study examined current practices, barriers, and readiness to implement screenings for food insecurity (FI), HPV risk, and vaccine recommendation.

**Methods:**

A cross‐sectional survey was conducted (August 2024) among Oklahoma dental hygienists. Using concepts from the Transtheoretical Model and the Theory of Planned Behavior (TPB), an embedded mixed‐methods survey evaluated respondent readiness to conduct FI screenings and HPV risk assessment/vaccine recommendation. Analyses included descriptive statistics and *χ* ^2^ tests to determine associations between intent and constructs of TPB. Additionally, content analysis of open‐ended questions identified barriers and facilitators to adopting these practices.

**Results:**

Among respondents (*n* = 92), the majority were not yet considering (precontemplation) screening for FI (67%), risk assessment for HPV (58%), or providing the HPV vaccine recommendation (78%). Most hygienists displayed a positive attitude toward these practices, yet lacked intent due to low perceived behavioral control and subjective norms (*p* < 0.001). Qualitative content analysis revealed barriers, including a lack of knowledge (41%) and awareness (18%), low confidence (10%), and considering such practices outside the scope of dental care (15%). Despite this, hygienists reported education (13%) and access to resources (10%) as facilitators in initiating HPV and FI related conversations.

**Implications:**

Despite the low readiness, hygienists reported a willingness to engage in these critical public health issues with appropriate training and support. A stage‐based training program in Oklahoma may enhance dental hygienists' knowledge and confidence, thereby improving preventive care and health outcomes.

## 1 Introduction

The scope of preventive dentistry has grown to address wider public health issues in addition to standard oral health care. An increasing awareness of the connection between oral and general health is reflected in current emphasis on more active integration of medical and dental aspects of healthcare. Patients, dental providers, and medical practitioners have all indicated support of such integration [1, 2, 3]. This empowers oral health professionals to consider their potential role in addressing complex public health problems not normally considered within the scope of practice. Such an interdisciplinary approach, referred to as medical dental integration (MDI), focuses on managing medical conditions by facilitating the coordination between dental and medical care [4]. By bridging these disciplines, oral health professionals can collaborate with medical professionals to identify certain medical conditions and foster a more broad and well‐coordinated approach to patient care. This inclusion within dental clinics offers a considerable opportunity for oral health professionals to identify individuals with undiagnosed conditions, particularly among those who do not seek routine general healthcare and provide referral [5].

In states like Oklahoma, which ranks among the lowest for healthcare access in the US [6], roles of oral health professionals become more relevant, placing them as key participants in promoting oral and overall health. Food insecurity and Human Papillomavirus infection (HPV) are two persistent public health issues with important oral health implications, both of which are often neglected in routine examinations. Additionally, they are both sensitive topics due to the intersection of complex health issues and socioeconomic challenges as it disproportionately affects those with decreased healthcare access, including low‐income and minoritized groups [7, 8]. These potentially uncomfortable topics require clinical judgment, communication skills, and attention to patient needs to avoid perpetuating the stigma [9]. Taking these two domains together allows an exploration of how oral health professionals conduct preventive practices in distinct, yet related, areas of care.

Food insecurity, as defined by the United States Department of Agriculture (USDA), refers to limited or uncertain access to enough food, often including worry about running out of food [10]. Chronic diseases, including a higher risk of cardiovascular disease mortality, often co‐occur with food insecurity, making it harder to maintain optimal systemic health. Notably, food insecurity disproportionately impacts households with low income, racial and ethnic minorities, and single mothers [10]. In 2023, 18 million United States (US) households (13.5%) experienced food insecurity, and 3.2 million of those homes had children who were food insecure [10]. Households that experience food insecurity are more likely to have diets that are low in nutrients and heavy in carbohydrates and sugars, in part due to significant barriers to healthy food access, which may affect oral health [11]. Notably, food insecurity has been found to increase the likelihood of untreated moderate and severe dental caries, suggesting a need for active involvement of dental professionals to mitigate factors contributing to and manage the consequences of food insecurity [11].

Another public health concern that disproportionately impacts people living in poverty and can cause oral health concerns is HPV [12]. HPV is a common viral infection, with an estimated incidence of 13 million people annually in the US, that usually affects the skin and mucous membranes. Concerningly, 79% of cancer cases in areas with HPV infection are likely related to the infection, with 40% of these being oropharyngeal cancers (OPC) [13]. Oral health professionals play an important role in the early detection of these cancers, identifying suspicious lesions of the oral mucosa and directing patients to specialists for further assessment, biopsy, and proper care. Oral health professionals can also play a role in the prevention of HPV through recommending the HPV vaccine, which can significantly reduce the risk of OPC and other HPV‐related cancers. The American Dental Association recommends that dental professionals take an active role in the recommendation of the HPV vaccination [14].

Given the wide‐ranging and substantial effects of food insecurity and HPV on oral and overall health, it would be beneficial for oral health professionals to conduct routine screening and actively recommend vaccination to eligible patients as part of their oral health management. This is especially important in states like Oklahoma, which have an increased prevalence of both food insecurity and HPV [15]. Considering the potentially sensitive nature of these public health issues, dental hygienists may be best equipped to address these with patients as they are more active in preventive oral care and often spend more time with patients through routine visits, building trust and establishing strong relationships with patients [9]. Despite this, little is known about dental hygienists' readiness and willingness to conduct such screenings. A better understanding of oral health professionals' readiness and perceptions of these screenings will inform the development of targeted interventions, such as continuing education (CE) courses, to support the implementation of food insecurity and HPV screenings in dental settings. Therefore, the purpose of this study was to explore the perceptions (i.e., current practices, barriers/facilitators, and readiness to adopt screenings) of dental hygienists in Oklahoma regarding medical screenings, with a focus on HPV risk assessment, HPV vaccination, and food insecurity screening. Exploring these two areas is ideal because they represent different types of practices—screening and providing behavioral recommendations. They also involve potentially stigmatizing health issues, so the barriers and facilitators identified are likely to also apply to less controversial topics, such as sugary drinks or tobacco.

## 2 Methods

An embedded mixed‐methods, cross‐sectional survey was conducted to explore the perceptions and current screening practices of dental hygienists related to food insecurity and HPV in Oklahoma. This study was deemed exempt by the Oklahoma State University Institutional Review Board in May 2024, and data were collected from June to August 2024. Because this was an exploratory study with an insufficient existing literature base to inform expected effect sizes, a priori power analysis and sample size estimation were not conducted.

### 2.1 Participants

The study participants included a convenience sample of dental hygienists. To be eligible, participants needed to be (1) licensed to practice in the US as dental hygienists, (2) currently practicing in the state of Oklahoma, and (3) self‐report regular, substantive interaction with patients to ensure inclusion of individuals who engage with fewer patients (e.g., part‐time providers or educators), but still engage directly in patient care, screening, and referrals. Participants who did not meet the eligibility criteria were excluded from the study. Consent was implied when participants proceeded beyond the information sheet presented at the beginning of the survey.

### 2.2 Survey Administration

To recruit participants, we partnered with the Oklahoma Dental Hygienists Association (OKDHA) and primarily disseminated the survey through their email newsletter, which reaches both members and non‐members. Over 2500 individuals received the email with an estimated 50% open rate. Additionally, we recruited participants through personal contacts and snowball sampling, whereby initial contacts were encouraged to share the survey link with other dental hygienists within their professional networks. The survey took approximately 15–20 min to complete, and participants received a $20 Amazon e‐gift card as compensation for their time. To protect participant confidentiality, contact details for reimbursement were collected in a separate survey.

### 2.3 Theories of Behavior Change

The Theory of Planned Behavior (TPB) and the Transtheoretical Model (TTM) offer complementary ways to understand why people engage in preventive behaviors and how oral healthcare professionals progress through stages when adopting new practices. The TPB is a framework used to understand an individual's attitudes and behaviors [16]. It predicts individual intention to engage in specific behaviors based on three fundamental factors: (1) behavioral beliefs reflect an individual's attitude toward performing a behavior, (2) social norms cover perceived social pressures to participate or abstain from the behavior, and (3) perceived behavioral control reflects an individual's beliefs about their capacity to act [16]. This relationship highlights the importance of intention in bridging the knowledge gap between attitudes and action in healthcare practices. Based on TPB, dental professionals' behaviors are influenced by their attitudes toward the importance of medical screenings (behavioral beliefs), the perceived social pressure or expectations from peers and patients to conduct these screenings (social norms), and their confidence in their ability to effectively perform these screenings (perceived behavioral control) [17].

The TTM suggests behavior modification is a gradual process involving stages: pre‐contemplation, contemplation, preparation, action, maintenance, and termination [18]. The model also helps understand the behaviors that can promote shifting between stages. A study on oral health practitioners' practices concerning eating disorders highlights the importance of TTM in identifying behavioral stages and obstacles, which helps direct interventions to improve preventive practices [19].

### 2.4 Measures

**FIGURE 1:**
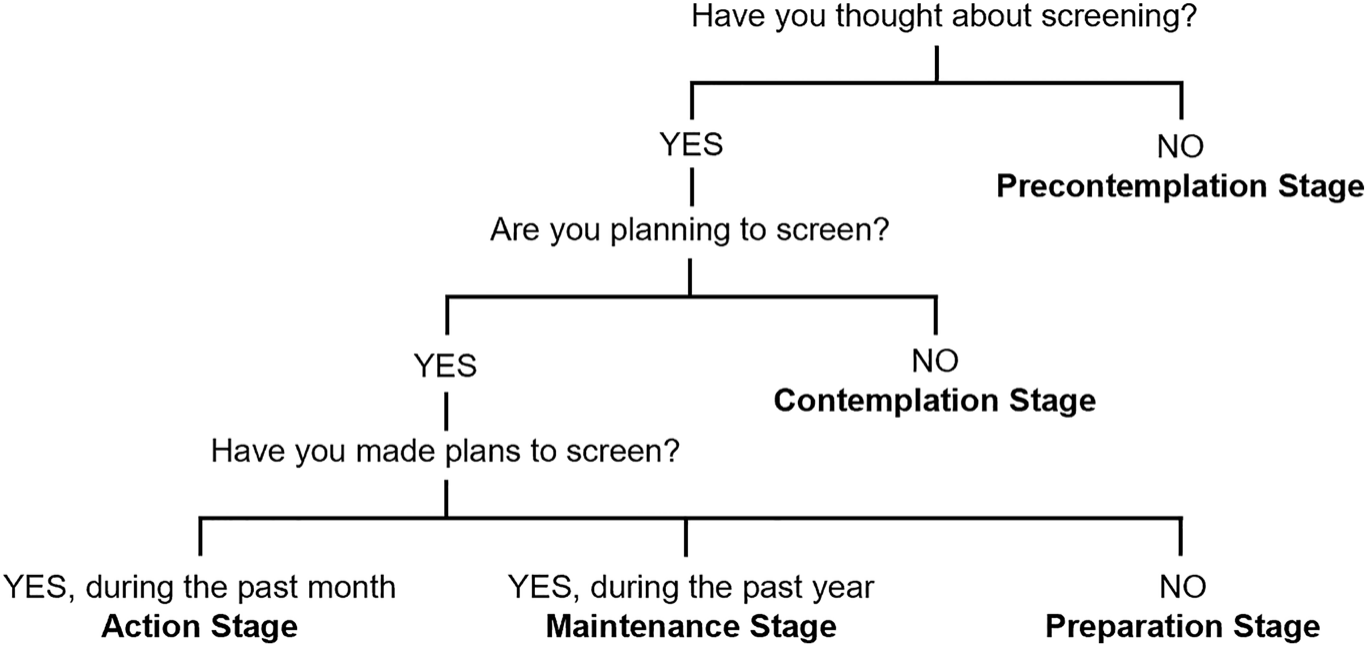
Survey questions based on the transtheoretical model. Adapted from Simons‐Morton and colleagues.

Participants reported sociodemographic information and professional characteristics (e.g., education, experience, occupation, location). Participants were also asked to estimate practice demographics (e.g., patient age, insurance coverage of patients). Survey items based on the TTM and TPB, which were adapted from previously validated items, are described in Figure 1 and Table 1, respectively [17, 20]. The TPB constructs were assessed using behavioral intention, attitude, subjective norms (patients and colleagues), and perceived behavioral control (i.e., confidence and ease). The TPB subscale for HPV risk assessment (*α* = 0.83), HPV vaccine recommendation (*α* = 0.84), and food insecurity screening (*α* = 0.82) demonstrated good internal consistency reliability. Participants also answered open‐ended questions about barriers and facilitators to conducting these screenings, based on whether or not they currently perform them.

**TABLE 1:** Theoretical constructs and corresponding survey questions.

Behavioral theory	Construct	Measure	Response format
Theory of planned behavior	Behavioral intent	Are you currently screening for…? HPV Infections Food insecurity	Dichotomous‐Yes/No
		Are you currently recommending the HPV vaccine to your eligible patients?	
	Attitude/anticipated outcome^a^	In the future, the dental professionals should have a role to play in…? HPV Screening Recommending HPV vaccination Food Insecurity screening	5‐point Likert scale‐strongly agree to strongly disagree
	Subjective norms^a^	Most of my dental colleagues provide prevention for all their patients HPV Screening Recommending HPV vaccination Food Insecurity screening	5‐point Likert scale‐strongly agree to strongly disagree
		My patients think as an Oral Healthcare Provider, I should use every opportunity to provide them with prevention HPV Screening Recommending HPV vaccination Food Insecurity screening	5‐point Likert scale‐strongly agree to strongly disagree
	Perceived behavioral control^a^	I feel confident about practicing ______ prevention if I wanted to HPV Screening Recommending HPV vaccination Food Insecurity screening	5‐point Likert scale‐strongly agree to strongly disagree
		For me to provide my patients with ______ is HPV Screening Recommending HPV vaccination Food Insecurity screening	5‐point Likert scale‐very easy to very difficult

^a^ Adapted from Yusuf and colleagues.

### 2.5 Data Analysis

Data analysis was conducted using IBM SPSS Statistics 29.0.1.0 (IBM, Chicago, IL) and R version 4.3.1 (R Core Team, Vienna, Austria). Demographic characteristics of the participants were summarized using descriptive statistics. A *χ* ^2^ test of independence was used to examine the association between behavioral intention, attitude, subjective norms, and perceived behavioral control related to HPV and food insecurity. Moreover, *χ* ^2^ tests were also used to determine the association between practice demographic characteristics and behavioral intent to engage in screenings. Fisher's exact test was utilized when cell counts were below five, as the assumptions for chi‐square were not met. Significance was set a priori at *p* = 0.00072, after applying a Bonferroni correction for multiple comparisons.

The responses collected from open‐ended questions were analyzed via deductive content analysis to identify key barriers and facilitators for practicing preventative screening [21]. Before analyzing the data, a codebook was created based on the body of existing literature. The coders were trained through review of the codebook, discussion of definitions, and calibration exercise. First, the first 25 responses were coded independently by two coders: the principal investigator (RL), who has experience in content analysis [22], and a graduate research assistant (TK). Minor adjustments were made to the codebook to improve coding consistency and the rigor of the analysis. (See Final codebook with coding definitions in Table 2). Second, the remaining responses were coded independently by both reviewers using the refined codebook. Coding differences were resolved to establish consensus. Overall percentage agreement between coders was 98.0%. The frequencies and percentages for each identified barrier and facilitator were computed by counting the occurrences across all responses.

**TABLE 2:** Summary of hygienist (*n* = 80) identified barriers and facilitators to conducting food insecurity screenings, HPV risk assessment and recommending the HPV vaccination.

Codes	% (n)	Definition	Exemplar quote
Barriers			
Knowledge	41.3% (33)	The provider does not have enough information, resources, and skills through education, training, or experience to address the topics	“I'm not educated enough to educate the patient on the subjects [HPV and food insecurity].”‐Hygienist 57 “Was not taught to offer that [vaccine recommendation] to patients.”‐Hygienist 58 “I need better advice on how to discuss this [HPV risk assessment and food insecurity] with patients.”‐Hygienist 62 “I do not feel like I have the accurate knowledge available. I know some about HPV vaccinations from myself, but not enough to share with others except for just very general information.”‐Hygienist 77
Awareness	17.5% (14)	The provider is not concerned or informed about these topics.	“I don't know what that [food insecurity] is and how it could relate to dentistry.”‐Hygienist 43 “I haven't had reason to believe it [food insecurity] was necessary but that's not to say it wasn't.”‐Hygienist 41
Confidence	10.0% (8)	The provider is uncomfortable due to a lack of knowledge or unsure about their ability to discuss these topics.	“If I had more education on both [HPV and food insecurity] then I would feel more comfortable discussing with patients.”‐Hygienist 14 “I just don't feel very confident discussing those [HPV and food insecurity] topics.”‐Hygienist 15
Privacy	10.0% (8)	Patients have the right to privacy and control over their personal information, yet discussions about these topics may lead to conflicts or disagreements between the patient and the provider regarding the use of this information.	“…patients seem like they are hesitant to share that [HPV and food insecurity] information because it is very personal to some of them.”‐Hygienist 4 “It feels uncomfortable‐difficult subjects [HPV and food insecurity] to breach.”‐Hygienist 69
Out of scope o practice	15.0% (12)	The topic for discussion is beyond the guidelines of a profession, current treatment plan, or clinic policy.	“Currently would only discuss food insecurity if there was something that raised that concern, but it is not a topic we bring up as a standard”‐Hygienist 28 “I didn't think much about it [HPV and food insecurity]/wasn't in regular [risk assessment and screening] routine”‐Hygienist 33 “I hadn't really thought about it [HPV vaccine recommendation] as being our [dental hygienists'] place”‐Hygienist 34
Non‐issue within practice setting	12.5% (10)	The issue is not a significant or relevant topic for the providers' practice setting.	“It [food insecurity] is not something we see often in our practice currently. It is not common among our patient pool.”‐Hygienist 28 “I feel in my practice it [HPV and food insecurity screening] is not necessary due to demographics”‐Hygienist 68
Time	6.25% (5)	There is not enough time during the patient visit/appointment to discuss these topics.	“Already have so much to do in so little time”‐Hygienist 49
Personal preferences	3.8% (3)	Providers' personal beliefs and preferences to discuss the topic.	“I don't really know if I'm interested in discussing [HPV] with patients.”‐Hygienist 72
Facilitators			
Education	12.5% (10)	The provider has enough knowledge and training to conduct a screening and discuss the topic.	“Learning about salivary testing and its role in oral health awareness”‐Hygienist 31 “Information from CE events”‐Hygienist 42 “Regarding HPV …, it's ingrained in me since hygiene school.”‐Hygienist 65
Access to resources	10.0% (8)	The providers have access to materials and tools to screen or refer patients for specific topics.	“Websites and handouts”‐Hygienist 55 “We give all patients a medical history form that also asks these questions so we can screen for all kinds of risks, insecurities, and habits.”‐Hygienist 56
Experience	8.8% (7)	The provider has prior exposure to knowledge and skills to perform screening and address the topic.	“Educating [patients] on how HPV affects the oral cavity/overall health… Also, asking open‐ended questions …similar challenges with food insecurity, and same approach.”‐Hygienist 36
Patient‐hygienist relation	3.8% (3)	A consensual agreement between the patient and healthcare provider based on trust and mutual respect.	“Being understanding/non‐judgmental has helped when advising patients…and building trust with patients.”‐Hygienist 36

## 3 Results

We received 112 responses, out of which five did not meet the eligibility criteria and 15 were incomplete. Table 3 represents demographic characteristics of all eligible respondents (*n* = 92).

**TABLE 3:** Demographic characteristics of survey respondents (*n* = 92).

Demographics	Response	Summary statistics
Median age % (*n*)	25–45 years	78.3% (72)
Gender % (*n*)	Female	98.9% (92)
	Male	1.1% (1)
Qualifications^a^ % (*n*)	BSDH	84.8% (78)
	AAS in DH	14.1% (13)
	Other	6.5% (6)
Experience % (*n*)	< 10 years	53.3% (49)
	> 10 years	46.7% (43)
Location % (*n*)	Medium Metro County	63.0% (58)
	Small Metro County	22.8% (21)
	Non‐metro or rural county	14.1% (13)
Patient Age^d^ Mean % ± SD	16–26 years	18.0 ± 9.0
	27–64 years	43.7 ± 15.5
	> 64 years	21.8 ± 13.4
Time spent with the patient in minutes Mean ± SD	Initial visit	77.9 ± 26.1
	Treatment visit	67.1 ± 26.1
	Follow‐up visit	52.4 ± 23.0
Insurance % Mean ± SD	Private insurance	66.6% ± 28.6
	Public assistance/none	37.8% ± 32.6
Practice setting^b^ % (*n*)	Private practice (owned)	62.0% (57)
	Private practice (associate)	17.4% (16)
	Community health center/public health unit	9.8% (9)
	Dental school clinics	7.6% (7)
	Other	7.6% (7)

*Note:* Location as defined by the National Center for Health Statistics Urban–Rural Classification Scheme. Percentages reflect estimates provided by dental providers regarding the age distribution of their patients. We calculated an average based on these estimates.

Abbreviations: AAS in DH, Associate of Applied Science in Dental Hygiene; BSDH, Bachelor of Science in Dental Hygiene.

^a^ Five respondents had more than one qualification.

^b^ Four respondents practiced in more than one setting type.

### 3.1 Demographics

Nearly all respondents identified as female (98.9%, *n* = 91) and held a Bachelor of Science in Dental Hygiene (BSDH, 84.5%, *n* = 78). Almost half (46.7%, *n* = 43) of the respondents had more than 10 years of experience as dental professionals, and the majority (63.0%, *n* = 58) came from medium metro counties. Dental hygienists spent the most time with patients during the initial/first visit (77.9 ± 26.1 min). Respondents worked in settings in which they estimated that one‐third of patients (37.8% ± 32.6%) had either no insurance or some form of public insurance, with the remainder having private insurance.

### 3.2 Communication About Non Oral Health Related Topics With Patients

#### 3.2.1 General

More than half of the hygienists (58.7%, *n* = 54) spent more than 5 min discussing health issues that affected oral health other than oral hygiene care. Among respondents, 76.1% (*n* = 70) reported that they are ‘usually’ or ‘always’ the ones responsible for addressing health topics outside of oral health within the practice, while 44.6% (*n* = 41) said these topics are ‘sometimes’ addressed by dentists.

### 3.3 Stage of Change for Preventive Practices

Respondents' stage of change for each preventive practice is displayed in Table 4.

**TABLE 4:** Distribution of dental providers across stages of change and the Theory of Planned Behavior constructs for HPV and food insecurity prevention in Oklahoma (*n* = 92).

		HPV risk assessment % (*n*)	HPV vaccine recommendation % (*n*)	Food insecurity screening % (*n*)
TTM stage of change				
Precontemplation		57.6% (53)	78.3% (72)	67.0% (63)
Contemplation		6.5% (6)	7.6% (7)	3.3% (3)
Preparation		10.9% (10)	1.1% (1)	12.0% (11)
Action		9.8% (9)	4.3% (4)	7.6% (7)
Maintenance		13.0% (12)	7.6% (7)	7.6% (7)
TPB construct and response				
Intent to implement screening	Yes	39.1% (36)	13.0% (12)	17.4% (16)
	No	59.8% (55)	85.9% (79)	82.6% (76)
Attitude toward screening	Agree	76.1% (70)	45.7% (42)	62.0% (57)
	Neutral	15.2% (14)	29.3% (27)	34.8% (32)
	Disagree	8.7% (8)	25.0% (23)	3.3% (3)
Subjective Norms‐Colleagues	Agree	30.4% (28)	14.1% (13)	20.7% (19)
	Neutral	28.3% (26)	23.9% (22)	39.1% (36)
	Disagree	41.3% (38)	62.0% (57)	40.2% (37)
Subjective Norms‐Patients	Agree	34.8% (32)	20.7% (19)	27.2% (25)
	Neutral	26.1% (24)	20.7% (19)	47.8% (44)
	Disagree	39.1% (36)	58.7% (54)	22.8% (21)
Perceived behavioral control‐confident in screening	Agree	46.7% (43)	27.2% (25)	38.0% (35)
	Neutral	13.0% (12)	19.6% (18)	27.2% (25)
	Disagree	39.1% (36)	53.3% (49)	34.8% (32)
Perceived behavioral control‐ease in screening	Easy	54.3% (50)	30.4% (28)	45.7% (42)
	Moderate	19.6% (18)	23.9% (22)	29.3% (27)
	Difficult	23.9% (22)	43.5% (40)	22.8% (21)

*Note:* The total number of responses does not add up to 92 because some participants selected “Prefer not to say.”

Abbreviations: TPB, theory of planned behavior; TTM, transtheoretical model.

#### 3.3.1 HPV‐Related

More than half the respondents had not considered (i.e., precontemplation stage) assessing for HPV risk (57.6%, *n* = 53), and 78.3% (*n* = 72) had not considered recommending the HPV vaccine. More hygienists were in the contemplation/preparation stages for HPV risk assessment (17.4%, *n* = 16) compared to vaccine recommendation (8.7%, *n* = 8). Similarly, 22.8% (*n* = 21) of respondents were currently assessing (i.e., action/maintenance stages) for HPV risk factors, but only 12.0% (*n* = 11) were recommending the vaccine.

#### 3.3.2 Food Insecurity

For food insecurity, two‐thirds of the respondents (67.0%, *n* = 63) had not considered screening. Furthermore, 15.2% (*n* = 14) of the respondents were in contemplation/preparation stages, and 15.2% (*n* = 14) were in action/maintenance stages.

### 3.4 Theory of Planned Behavior Constructs for Preventive Practice

Table 4 provides a summary of the participant responses for each preventative practice for TPB constructs. Table 5 highlights the association between intent, attitude, subjective norms, and perceived behavioral control for each practice. Overall, the study found statistically significant (*p* < 0.0007) differences across all measures for HPV risk assessment and vaccine recommendation, except for subjective norms. Higher levels of behavioral intent were linked to positive outcomes.

**TABLE 5:** Association between intent, attitude, subjective norms, and perceived behavioral control for medical screenings (*n* = 92).

Behavior	Constructs^a^	Response	Intent		
			Yes % (n)	No % (n)	
HPV risk assessment			*n* = 36	*n* = 55	
	Attitude^b^	Agree	97.2% (35)	61.8% (34)	*
		Neutral	2.8% (1)	23.6% (13)	
		Disagree	0.0% (0)	14.5% (8)	
	Subjective Norms‐Colleagues	Agree	58.3% (21)	10.9% (6)	*
		Neutral	25.0% (9)	30.9% (17)	
		Disagree	16.7% (6)	58.2% (32)	
	Subjective Norms‐Patients	Agree	47.2% (17)	27.3% (15)	
		Neutral	33.3% (12)	20.0% (11)	
		Disagree	19.4% (7)	52.7% (29)	
	Perceived Behavioral Control‐Confidence^b^	Agree	80.6% (29)	23.6% (13)	*
		Neutral	5.6% (2)	18.2% (10)	
		Disagree	13.9% (5)	56.4% (31)	
	Perceived Behavioral Control‐Ease	Easy	80.6% (29)	36.4% (20)	*
		Moderate	11.1% (4)	25.5% (14)	
		Difficult	5.6% (2)	36.4% (20)	
HPV vaccine recommendation			*n* = 12	*n* = 79	
	Attitude^b^	Agree	100.0% (12)	38.0% (30)	*
		Neutral	0.0% (0)	34.2% (27)	
		Disagree	0.0% (0)	27.8% (22)	
	Subjective Norms‐Colleagues^b^	Agree	66.7% (8)	6.3% (5)	*
		Neutral	8.3% (1)	25.3% (20)	
		Disagree	25.0% (3)	68.4% (54)	
	Subjective Norms‐Patients^b^	Agree	50.0% (6)	16.5% (13)	
		Neutral	16.7% (2)	21.5% (17)	
		Disagree	33.3% (4)	62.0% (49)	
	Perceived Behavioral Control‐Confidence^b^	Agree	100.0% (12)	16.5% (13)	*
		Neutral	0.0% (0)	21.5% (17)	
		Disagree	0.0% (0)	62.0% (49)	
	Perceived Behavioral Control‐Ease^b^	Easy	83.3% (10)	22.8% (18)	*
		Moderate	8.3% (1)	26.6% (21)	
		Difficult	0.0% (0)	49.4% (39)	
Food insecurity screening			*n* = 16	*n* = 76	
	Attitude^b^	Agree	93.8% (15)	55.3% (42)	
		Neutral	6.3% (1)	40.8% (31)	
		Disagree	0.0% (0)	3.9% (3)	
	Subjective Norms‐Colleagues^b^	Agree	56.3% (9)	13.2% (10)	*
		Neutral	25.0% (4)	42.1% (32)	
		Disagree	18.8% (3)	44.7% (34)	
	Subjective Norms‐Patients^b^	Agree	56.3% (9)	21.1% (16)	
		Neutral	31.3% (5)	51.3% (39)	
		Disagree	12.5% (2)	25.0% (19)	
	Perceived behavioral control‐confidence^b^	Agree	81.3% (13)	28.9% (22)	*
		Neutral	12.5% (2)	30.3% (23)	
		Disagree	6.3% (1)	40.8% (31)	
	Perceived behavioral control‐Ease^b^	Easy	68.8% (11)	40.8% (31)	
		Moderate	25.0% (4)	30.3% (23)	
		Difficult	6.3% (1)	26.3% (20)	

^a^ Constructs reflect those in theory of planned behavior.

^b^ Fisher's exact test was used in cases where at least one expected cell count was below 5 to ensure the accuracy of the statistical analysis in the *χ* ^2^ table.

* Denotes significance at Bonferroni corrected *p*‐value of 0.00072.

#### 3.4.1 HPV Risk Assessment

Almost all individuals who had behavioral intention to screen agreed (97.2%, *n* = 35) that HPV screening was relevant in dental settings compared to 61.8% (*n* = 34) among those who did not intend to screen. A significant number of respondents who planned to screen had much higher confidence (80.6%, *n* = 29) and were more likely to favor ease of performing screenings (80.6%, *n* = 29) than those who had no plans (*p* < 0.0007).

#### 3.4.2 HPV Vaccination Recommendation

Each respondent who had a behavioral intent to recommend the HPV vaccine believed that hygienists are important in preventing HPV (100%, *n* = 12) compared to 54.5% (*n* = 30, *p* < 0.0007) of those who did not intend to recommend it. In addition, all respondents with behavioral intention to recommend the vaccine were confident (100.0%, *n* = 12) in their ability to do so, while a significant number of the hygienists with no intentions had no confidence (62.0%, *n* = 49) in recommending the vaccine (*p* < 0.0007).

#### 3.4.3 Food Insecurity

Compared to those without a behavioral intent to screen for food insecurity (28.9%, *n* = 22), those with the intention (81.3%, *n* = 13) were far more likely to feel confident in carrying out food insecurity screening (*p* < 0.0007). Notably, more than half of respondents (56.3%, *n* = 9) intending to screen for food insecurity believed that their colleagues actively engage in screening, while only 13.2% (*n* = 10) without intent agreed (*p* < 0.0007).

The majority of respondents (87.0%, *n* = 80) mentioned at least one facilitator and/or barrier associated with food insecurity screening, vaccine recommendations, and HPV screening. Overall, eight barriers and four facilitators to offering preventative screenings were identified. The barriers/facilitators are summarized in Table 2, along with definitions and exemplar quotes.

### 3.5 Barriers

#### 3.5.1 Knowledge

The most commonly mentioned barrier among respondents was a lack of knowledge (41.3%, *n* = 33) about HPV and food insecurity. This manifested in several ways, including a lack of topical knowledge and necessary training. One hygienist, indicating a lack of general knowledge, reported “*not knowing enough information on these two topics [HPV and food insecurity] to discuss them with patients*.” Similarly, another hygienist described a lack of training on appropriate screening protocols as not knowing “*how to implement a screening [for HPV and food insecurity].*” Some felt they needed more training on the resources available to patients who screened positive. One hygienist asked, “*what resources we could even provide for this [food insecurity]?*”

#### 3.5.2 Awareness

Another commonly cited barrier was a lack of awareness (17.5%, *n* = 14) of the topics. This primarily manifested as being unaware of the definition of food insecurity or never considering implementing these screenings into their practice. Notably, several hygienists reported being “*not sure what that [food insecurity] is and how it could relate to dentistry.*” Several respondents also reported that they have “*never thought*” or “*never considered*” integrating food insecurity or HPV screening in their practice.

#### 3.5.3 Confidence

Some hygienists also specifically reported lacking confidence (10.0%, *n* = 8) to address these issues as a key barrier. Hygienists “*don't feel comfortable*” or “*don't feel confident enough knowledge‐wise*” in addressing HPV and food insecurity. This also connected back to knowledge, as this hygienist reported, “*little information on the topic [makes it challenging] to feel comfortable advising a patient clinically.*”

#### 3.5.4 Privacy

A further significant concern was privacy (10.0%, *n* = 8) and the controversial nature of these topics. Hygienists expressed the possibility of having “*awkward*” conversations, leading to patients feeling these topics are “*too personal*” to discuss in a clinic setting or “*judged*” by their hygienist. Vaccines were particularly viewed as sensitive, with one respondent calling them a “*touchy topic*” and another noting, “*it is my duty to inform patients but recommending them…is not within my comfort zone.*” Privacy concerns, particularly around HPV vaccines, also manifested as a “*fear of upsetting the patient*” among some hygienists.

#### 3.5.5 Scope of Practice

An additional recurrent theme was the misperception of food insecurity and HPV in relation to oral care, with several hygienists reporting these issues were inconsistent with their role scope (15.0%, *n* = 12). For some, this manifested as being outside their scope of practice, whereas for others, they perceived that patients would feel these topics are outside the scope of routine dental care. While some hygienists felt that vaccines “*should be discussed with a primary care physician first*” and belong under medical care rather than dental practice, this was less common than concerns around patient perceptions. In reference to food insecurity, one oral healthcare professional stated that although diet is important for oral health, “*patients may feel it is outside the scope of a dental visit*.” This attitude was supported by practice norms; for example, a faculty member at the dental hygiene school noted that screening “*is not in our current treatment plan for students.*” For several hygienists, this manifested as a reactive approach to screening, where the provider does not “…*think about it unless the patient is concerned*.”

#### 3.5.6 Non‐Issue Within Practice Setting

Some hygienists perceived screening as unnecessary, believing their patient populations were unlikely to be affected (12.5%, *n* = 10). For example, respondents in self‐described high‐income, private practices assumed food insecurity was irrelevant to their population, stating “*we work in a fee‐for‐service office…[food insecurity] isn't something we see.*” Similarly, HPV was often seen as irrelevant to older adults, as one hygienist noted, “*[they] mainly see an older population.*”

#### 3.5.7 Time and Personal Preferences

Another barrier noted among a few hygienists was limited time during appointments (6.25%, *n* = 5). This primarily manifested as a hesitancy to take on additional responsibilities amid a pressure to “*stay caught up.*” Another barrier noted among a subset of respondents (3.8%, *n* = 3) was prioritizing other issues above these screenings or personal views that shaped their decisions, such as believing these topics were “*not as important [as other topics].*”

### 3.6 Facilitators

#### 3.6.1 Education

The most commonly reported facilitator was being educated (12.5%, *n* = 10) on the topics. Respondents felt confident in engaging on sensitive topics with the patients due to their prior “*studies*” and “*training*.” Several individuals stressed that their primary sources of knowledge were “*continuing education classes*” and the “*dental/oral health information [they] have acquired*” over time, which improved their understanding of the topic.

#### 3.6.2 Access to Resources

Some hygienists also noted having access to tools and resources (10.0%, *n* = 8) as a facilitator that enables them to confront delicate subjects and motivate patients to change their behavior. To aid in these screenings, some dental hygienists used clinical instruments such as “*intraoral photos, full medical histories, and medication interactions*” and “*positive biopsy results to diagnose HPV.*” Others emphasized the value of “*literature for the patient [related to these topics]*” and “*knowledge of local resources available*” when speaking with patients about HPV and food insecurity.

#### 3.6.3 Experience

Another significant element that gave hygienists the confidence to address issues like HPV and food insecurity was prior experience (8.8%, *n* = 7). Through repeated “*practice*” and “*patient experiences*,” hygienists gained a clearer understanding of how to apply their knowledge, recognizing the “*risks and benefits*” in real‐world situations and the skills to approach these sensitive conversations with patients.

#### 3.6.4 Patient‐Hygienist Relation

Additionally, the strength of the relationship between the patient and the oral health professional was relevant to some hygienists (3.8%, *n* = 3). According to the respondents, “*building trust*” and “*routine oral cancer screenings*” helped patients become more receptive to advice, as they “*build value and patients feel taken care of.*” Patients were also encouraged to talk openly when oral health professionals approached conversations with an “*understanding/non‐judgmental*” attitude, especially when discussing subjects that may be stigmatized. Lastly, the dental hygienist's willingness to “*educate patients*” was perceived as helpful in promoting discussions about these subjects.

## 4 Discussion

The study examined dental hygienists' perceptions of HPV risk assessment, vaccine recommendation, and food insecurity screenings. While hygienists acknowledged the importance of these practices, their behavioral intention to adopt them remained low. Barriers identified included a lack of knowledge, low confidence, and viewing these practices as beyond their scope. These findings suggest the need for stage‐based CE programs that guide participants from awareness to adoption of preventive practices.

The findings from our study indicate that there is an attitude‐intention gap among dental hygienists regarding both HPV and food insecurity prevention. The lack of translation of a positive attitude into behavioral intent in the current study is consistent with the literature, suggesting that several barriers prevent favorable attitudes from resulting in action [23]. Although somewhat understudied in this context, the attitude‐intention gap observed in the current study is widely studied in behavioral research [24], and several studies reported similar intrapsychic and contextual factors. Positive attitudes are still essential for behavior change, but intrapsychic barriers such as low perceived behavioral control, perceived role, and self‐efficacy contribute to low intent [25]. This reluctance is exacerbated by contextual factors such as those barriers noted in this and other studies: time limitations, insufficient support within clinic systems, hierarchical relations with dentists and clinic‐level policies [26]. To effectively close the attitude‐intention gap, future research should focus on identifying ways to address both intrapsychic and contextual barriers within education interventions with hygienists.

Individual, professional, and systemic factors all have an impact on preventive screenings in dental settings. Hygienists indicated a lack of understanding and awareness about addressing HPV and food insecurity in practice, which is consistent with prior research relating a lack of awareness to low adoption of preventive practices [25]. While most acknowledged that screening for food insecurity is important, dental hygienists pointed to a lack of training as a hindrance. Although the link between food insecurity and oral health, particularly dental caries, is widely acknowledged, misunderstandings about definitions, implications, and accessible services have eroded oral health professionals' confidence [27]. These findings point to the need for CE programs to address basic knowledge gaps before implementing screening protocols.

Education and training were identified by healthcare professionals as key to boosting their confidence and ability to implement screening practices, reinforcing prior findings that training is critical for driving behavior change, leading to implementing new practices [28]. Our findings are consistent with prior research demonstrating that successful MDI is facilitated when healthcare professionals are provided with sufficient resources and support in overcoming barriers. Healthcare professionals with screening tools and referral pathways are more likely to address food insecurity, and those with vaccine communication training are more likely to talk about HPV vaccination [28]. These results emphasize the value of communication training in addition to knowledge in assisting professionals in handling delicate subjects. In addition to knowledge gaps, several health professionals mentioned time constraints as a barrier, though this was less frequent than in previous studies where divergent needs and active schedules were major issues [29]. Healthcare professionals' understanding of the screening process may influence their perception of time, and integrating screenings into routine workflows could help alleviate this burden [30]. Educating hygienists on best practices for integrating screenings into routine workflows may empower them to champion these practices in their clinics, which may increase clinic uptake, thereby strengthening connection between medical and dental services [28].

Successful integration also necessitates overcoming barriers, especially those pertaining to patient privacy and controversial topics, which may impact patient and oral health professional participation. Concerns regarding patient privacy and discomfort with delicate subjects like HPV are prevalent in the literature across healthcare settings [7]. Although healthcare professionals frequently worry about upsetting their patients, strong patient‐hygienist/provider relationships, as in this and other studies, may help make these discussions easier [31]. The significance of these relationships in screening uptake is highlighted by the fact that patients are more likely to accept preventive therapies when they get recommendations from trusted healthcare professionals [32]. However, screening alone is insufficient; oral health professionals must also have clear instructions and tangible resources to address food insecurity effectively and sensitively.

The interpretation and generalizability of the results of this study are impacted by a number of limitations. Because the study was limited to Oklahoma, its findings might not apply to areas with distinct demographic or cultural characteristics. Moreover, the Oklahoma Dental Hygienists Association newsletter served as the primary recruitment tool for the study, which restricted the sample to individuals who were more actively involved in the profession as opposed to those who were not. Notably, including only dental hygienists' perspectives might have reduced the comprehensiveness of the findings and limited their applicability to all oral health professionals. Also, the small sample size may have constrained the ability to identify variations between subgroups based on years of experience, rurality, or the primary insurance type of patients. Furthermore, there is a chance of social desirability bias when using self‐reported data since participants might have given responses they thought were anticipated rather than completely truthful. Beyond this, it is possible that limiting the open‐ended responses to facilitators for doers and barriers for non‐doers may have underestimated a range of factors impacting behaviors. These limitations emphasize the necessity of more extensive, varied sample sizes in subsequent studies to validate and expand upon these outcomes. Despite these limitations, the findings of this study provide important insights into Oklahoma hygienists' willingness to conduct these key preventative health screenings and the barriers and facilitators they experience.

## 5 Implications

CE is critical to building knowledge, skills, and confidence in the integration of preventive care, including vaccine recommendations and medical screening. While it can improve knowledge, research indicates that CE does not effectively address confidence and behavioral change [33], especially regarding sensitive topics like HPV and food insecurity. While traditional didactic CE can increase awareness, it may be unlikely to meaningfully reduce discomfort or improve communication skills. Griner and colleagues found that CE greatly improved dental hygienists' knowledge of HPV‐related subjects, but had no impact on their confidence in their ability to advise patients or propose vaccines [33]. Similarly, for food insecurity, educational courses increased knowledge but did not eliminate discomfort in discussing it with patients among medical students [28], whereas courses that focused on experiential learning showed higher confidence and improved referral abilities [34]. Furthermore, CE models are often standardized; they may not consider the readiness to change, which may be influenced by intrapsychic and contextual barriers. In order to bridge these barriers, CE courses should be tailored, practical, and integrated into routine professional development to help dental hygienists successfully adopt preventive screening practices with confidence. This approach can foster better MDI, where oral health professionals recommend vaccines and encourage patients to seek medical care for identified risks. Once oral health professionals are prepared, having clear referral pathways will facilitate patient awareness of subsequent steps in their care, enhancing adherence to preventive health interventions.

## Funding

This work was supported by Oklahoma State University College of Education and Human Sciences.

## Conflicts of Interest

The authors declare no conflicts of interest.

## Data Availability

The data that support the findings of this study are available from the corresponding author upon reasonable request.
